# Supplementary material for: Content-rich biological network constructed by mining PubMed abstracts
Source: BMC Bioinformatics. 2004 Oct 8;5:147. doi: 10.1186/1471-2105-5-147 (PMC528731; doi:10.1186/1471-2105-5-147)
Supplement: Additional File 2 — The original results of the above study (non-essential files are deleted to keep the file size under the limit set by BMC bioinformatics). [file 1471-2105-5-147-S2.bz2 › chilibotAdditionalFile2/dip05/506ID11278309E10794/html/AR_CAV1.html]

 


 **AR** and **CAV1** 
  
Found 18 abstracts in PubMed, retrieved 05.  
 

 What does Google say? 
 PDF only 
| .edu only 

---

**Interactive relationship** (e.g. stimulation, inhibition, etc)

**Inhibitory relationship**- The sequestration of alpha2A  **AR**  was partly inhibited after sucrose pretreatment but could be further reduced after inhibiting the formation of both clathrin coated and caveolin  [ **CAV1** ]  vesicles by combined pretreatment with hyperosmotic sucrose and filipin.  Ref: 12800978 Eur J Cell Biol, 2003
**Neutral relationship**- Antisense and pharmacological experiments provided additional evidence that the sequential activation of PKCepsilon, mitogen activated protein kinases, c Myc, and androgen receptor  [ **AR** ]  signaling drove the downstream expression of caveolin 1  [ **CAV1** ]  in CWR R1 cells.  Ref: 12185081 J Biol Chem, 2002

**Non-interactive relationship** (e.g. studied together, co-existance, homology, etc.)

- Caveolin 1  [ **CAV1** ] , androgen receptor  [ **AR** ] , c Myc, and protein kinase Cepsilon PKCepsilon proteins are overrepresented in most advanced prostate cancer tumors.  Ref: 12185081 J Biol Chem, 2002
